# *CIAO1* loss of function causes a neuromuscular disorder with compromise of nucleocytoplasmic Fe-S enzymes

**DOI:** 10.1172/JCI179559

**Published:** 2024-06-17

**Authors:** Nunziata Maio, Rotem Orbach, Irina T. Zaharieva, Ana Töpf, Sandra Donkervoort, Pinki Munot, Juliane Mueller, Tracey Willis, Sumit Verma, Stojan Peric, Deepa Krishnakumar, Sniya Sudhakar, A. Reghan Foley, Sarah Silverstein, Ganka Douglas, Lynn Pais, Stephanie DiTroia, Christopher Grunseich, Ying Hu, Caroline Sewry, Anna Sarkozy, Volker Straub, Francesco Muntoni, Tracey A. Rouault, Carsten G. Bönnemann

**Affiliations:** 1 Molecular Medicine Branch, Eunice Kennedy Shriver National Institute of Child Health and Human Development (NICHD), National Institutes of Health (NIH), Bethesda, Maryland, USA.; 2 Neuromuscular and Neurogenetic Disorders of Childhood Section, National Institute of Neurological Disorders and Stroke (NINDS), NIH, Bethesda, Maryland, USA.; 3 Dubowitz Neuromuscular Centre, UCL Great Ormond Street Institute of Child Health, London, United Kingdom.; 4 John Walton Muscular Dystrophy Research Centre, Translational and Clinical Research Institute, Newcastle University and Newcastle Hospitals NHS Foundation Trust, Newcastle upon Tyne, United Kingdom.; 5 Wolfson Centre for Neuromuscular Disorders, Robert Jones and Agnes Hunt Orthopaedic Hospital, Oswestry, United Kingdom.; 6 Chester University Medical School, Chester, United Kingdom.; 7 Department of Pediatrics and Neurology, Emory University School of Medicine, Georgia, Atlanta, USA.; 8 Department for Neuromuscular Disorders, Neurology Clinic, University Clinical Centre of Serbia, Faculty of Medicine, University of Belgrade, Belgrade, Serbia.; 9 Paediatric Neurology, Addenbrooke’s Hospital, Cambridge University Hospitals NHS Foundation Trust, Cambridge, United Kingdom.; 10 Department of Neuroradiology, Great Ormond Street NHS Trust Hospital, London, United Kingdom.; 11 GeneDx, LLC, Gaithersburg, Maryland, USA.; 12 Program in Medical and Population Genetics, Center for Mendelian Genomics, Broad Institute of MIT and Harvard, Cambridge, Massachusetts, USA.

**Keywords:** Metabolism, Muscle biology, DNA repair, Genetic diseases

## Abstract

Cytoplasmic and nuclear iron-sulfur (Fe-S) enzymes that are essential for genome maintenance and replication depend on the cytoplasmic Fe-S assembly (CIA) machinery for cluster acquisition. The core of the CIA machinery consists of a complex of CIAO1, MMS19 and FAM96B. The physiological consequences of loss of function in the components of the CIA pathway have thus far remained uncharacterized. Our study revealed that patients with biallelic loss of function in *CIAO1* developed proximal and axial muscle weakness, fluctuating creatine kinase elevation, and respiratory insufficiency. In addition, they presented with CNS symptoms including learning difficulties and neurobehavioral comorbidities, along with iron deposition in deep brain nuclei, mild normocytic to macrocytic anemia, and gastrointestinal symptoms. Mutational analysis revealed reduced stability of the variants compared with WT CIAO1. Functional assays demonstrated failure of the variants identified in patients to recruit Fe-S recipient proteins, resulting in compromised activities of DNA helicases, polymerases, and repair enzymes that rely on the CIA complex to acquire their Fe-S cofactors. Lentivirus-mediated restoration of *CIAO1* expression reversed all patient-derived cellular abnormalities. Our study identifies *CIAO1* as a human disease gene and provides insights into the broader implications of the cytosolic Fe-S assembly pathway in human health and disease.

## Abstract 2

Patients with loss of function in CIAO1, which is required to maintain genome integrity, experience multisystem involvement, including muscle weakness, respiratory insufficiency, and neurological comorbidities.

## Graphical abstract

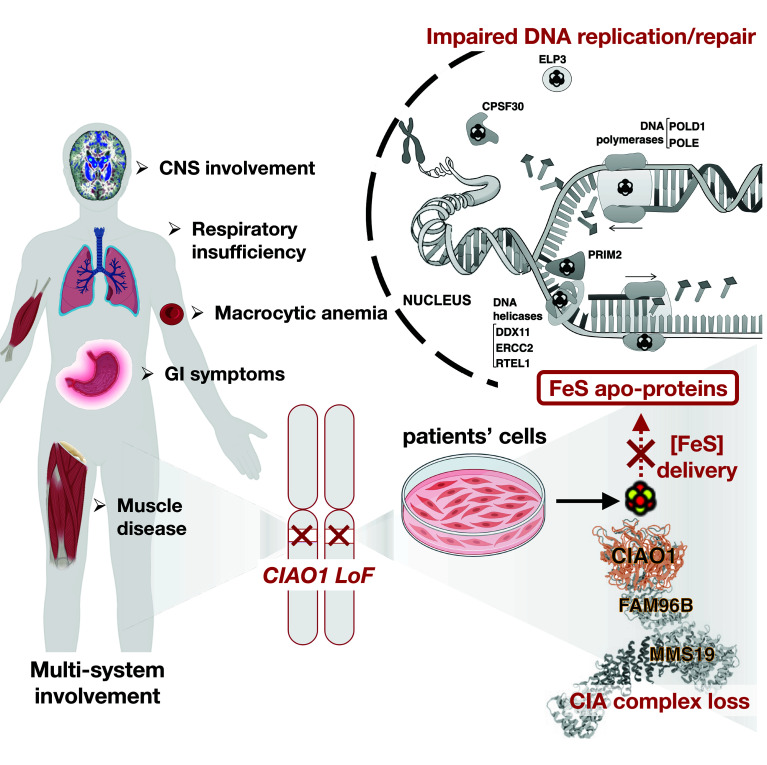

## Introduction

**Figure 1:**
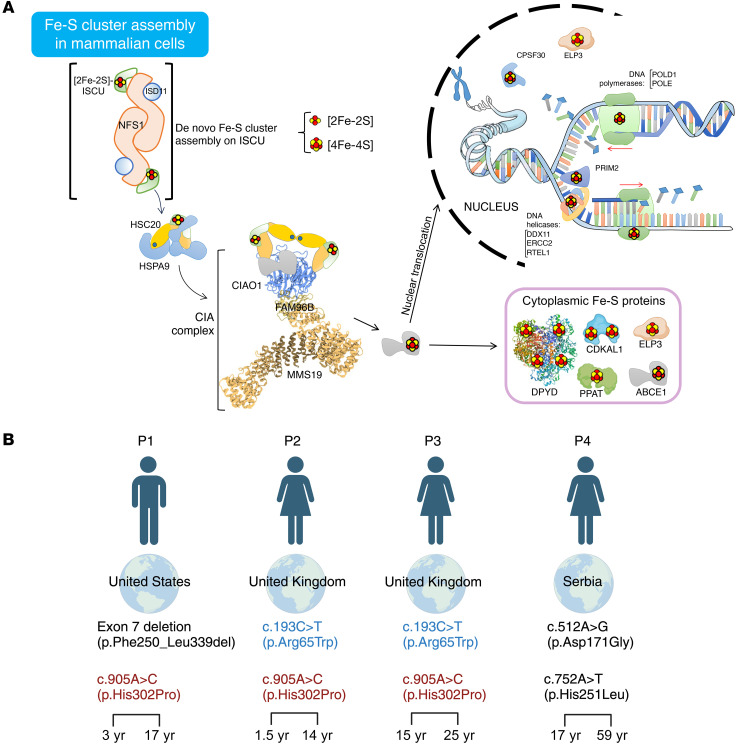
Identification of biallelic *CIAO1* variants in 4 independent patients with a neuromuscular condition of undefined etiology. (**A**) Proposed model for the biogenesis of ISCs in mammalian cells. De novo assembly of ISCs occurs upon the main scaffold protein ISCU by the coordinated action of a multiprotein complex, which consists of the cysteine desulfurase NFS1 and the accessory protein ISD11. The HSC20-HSPA9 cochaperone-chaperone complex interacts with ISCU to facilitate cluster transfer to recipient proteins. The functional unit of HSC20 is a dimer (15). A subset of recipient Fe-S proteins acquire their clusters directly from the HSC20-HSPA9-ISCU1 complex (15). In the cytoplasm, binding of HSC20 to the LYR motif of CIAO1 recruits the CIA-targeting complex, which is known to form a platform to which Fe-S recipients involved in DNA metabolism dock to acquire their clusters (13, 14). The Fe-S proteins shown in the model were all identified as HSC20 interacting partners (15) (i.e., NUBP2, GLRX3, CIAPIN1, ABCE1, ERCC2, POLD1, PRIM2, PPAT, ELP3, CPSF30, DDX11, etc.). (**B**) Diagram showing the country of origin of the 4 patients with biallelic *CIAO1* variants. Recurring variants are shown in red and blue. The age when symptoms were first recognized (on the left) and the age at the latest clinical assessment (on the right) are shown at the bottom. Figure 1B was created with BioRender.com.

Iron-sulfur (Fe-S) clusters are ancient and evolutionarily conserved prosthetic groups with unique chemical properties that enable the proteins that contain them (Fe-S proteins) to function in several essential cellular pathways, including DNA replication and repair, ribosome biogenesis and protein translation, biosynthesis of heme and essential cofactors, such as lipoic acid, electron transfer in the mitochondrial respiratory chain, and substrate coordination in dehydratases such as mitochondrial aconitase of the citric acid cycle (1). Biogenesis of all Fe-S proteins depends on the core Fe-S cluster (ISC) assembly machinery (Figure 1A) (1, 2). In mammalian cells, ISCs are assembled de novo by a complex composed of a cysteine desulfurase, NFS1, its binding partner, LYRM4 (also known as ISD11), the acyl carrier protein NDUFAB1, the initial assembly scaffold, ISCU, an allosteric effector, frataxin, and ferredoxin and ferredoxin reductase, a source of reducing equivalents (3, 4). Upon assembly of a nascent cluster, the scaffold protein ISCU binds to an evolutionarily highly conserved co-chaperone/chaperone system, consisting of HSC20 (or HSCB) and HSPA9, respectively, in humans (Figure 1A) (5, 6). The HSC20-HSPA9 complex enhances ISC transfer from the main scaffold ISCU directly to recipient proteins or to secondary carriers, which then deliver Fe-S cofactors to recipient apoproteins. Importantly, the core mammalian ISC components have been previously identified in both the mitochondrial matrix and the cytosol and nucleus, where they operate in parallel to assemble ISCs in the subcellular compartments of multicellular eukaryotes (5, 7–12).

A subset of cytoplasmic and nuclear Fe-S enzymes requires the specialized cytoplasmic Fe-S assembly (CIA) complex, composed of CIAO1, MMS19, and FAM96B, which acts downstream of the ISC system, to acquire their cofactors (Figure 1A) (13–15). CIAO1 is a key component of the CIA machinery (13–15). The functional consequences of loss-of-function variants in *CIAO1* or in any of the CIA components have thus far remained unknown.

In this study, we identified 4 unrelated patients with biallelic loss of function in *CIAO1*, who presented with a distinctive syndrome of predominantly neuromuscular manifestations and a spectrum of multisystem findings. Biochemical and functional studies in patient-derived cell lines and tissues confirmed the pathogenicity of the variants and enabled the molecular characterization of the phenotype associated with loss of function in *CIAO1* characterized by the compromise of nucleocytoplasmic Fe-S enzymes involved in genome replication and maintenance, transfer RNA (tRNA) modification, and purine and pyrimidine metabolism. Lentivirus-mediated restoration of *CIAO1* expression reverted all the abnormalities of the patient-derived cells, thereby demonstrating that loss of function of CIAO1 caused the phenotype observed in the patients. Together, our studies define the critical requirement of *CIAO1* for human health and physiology and collectively contribute to a better understanding of the consequences of loss of function in a key component of the CIA pathway necessary for genome replication and maintenance.

## Results

Loss of function in nearly all ISC assembly components has emerged over the past decade as the leading cause of multiple rare human conditions (16). However, thus far, deficiencies in the core components of the CIA complex, composed of CIAO1, MMS19, and FAM96B, have not been reported. In this study, we present the cases of 4 genetically unrelated individuals with loss of function in *CIAO1* (herein named P1, P2, P3, and P4).

### Genetic findings

In all 4 families, extensive next-generation–based sequencing identified no pathogenic variants in the known human disease genes evaluated with adequate coverage. Upon further analysis of exome-sequencing data, biallelic variants in *CIAO1* (NM_004804.3) were identified (Figure 1B). Segregation testing, when available (P1, P2), was consistent with autosomal recessive inheritance. Two missense variants were recurrent: p.His302Pro (*n* = 3, in P1, P2 and P3) and p.Arg65Trp (*n* = 2, in P2 and P3). Two variants were found in compound heterozygosity in P4 (p.Asp171Gly; p.His251Leu). P1 was compound heterozygous for a maternally inherited recurring missense variant (p.His302Pro) and a paternally inherited deletion of exon 7 (p.F250_L339del) of *CIAO1* (Figure 1B), resulting in an out-of-frame transcript predicted to undergo nonsense-mediated decay. This was confirmed by RNA-Seq on RNA extracted from dermal fibroblasts, which revealed that P1 was apparently “homozygous” for the maternal *CIAO1* allele, with no evidence of paternal reads (Supplemental Figure 1, A and B; supplemental material available online with this article; https://doi.org/10.1172/JCI179559DS1). The Sashimi plot showed reduced *CIAO1* expression in fibroblasts from P1 compared with 3 aggregated control samples, with no effect on splicing (Supplemental Figure 1C).

The 4 *CIAO1* missense variants that were predicted to alter a conserved single amino acid were only found in heterozygosity in gnomAD (SVs version 2.1) (17). Variants were predicted to be disease causing by various in silico computational predictions (Supplemental Table 1).

### Clinical presentation

All 4 patients had onset of weakness in early childhood to the adolescent years (1.5–17 years) (Figure 1B). Detailed clinical information is included in Table 1. Core phenotypic features in all patients, consistent with the presence of a myopathy with dystrophic features, included slowly progressive muscle weakness of proximal and axial skeletal muscles, mild facial and bulbar weakness, respiratory insufficiency (*n* = 4, forced vital capacity [FVC] 51%–63% predicted), fatigability/low endurance (*n* = 4), joint hyperlaxity (*n* = 4), ankle tightness (*n* = 4), calf pseudohypertrophy (*n* = 3, not commented for P2), and elevated serum creatine kinase (CK) levels (*n* = 4) (Table 1). Findings pointing to CNS and multisystem involvement included learning disabilities/difficulties (*n* = 4), neurobehavioral comorbidities (*n* = 2), normocytic-to-macrocytic anemia (*n* = 2), constipation and gastrointestinal (GI) symptoms (*n* = 2), obstructive sleep apnea (*n* = 2), and overweight (*n* = 3). The 4 patients underwent standard cardiology evaluations, encompassing ECG and echocardiography, which were essentially unremarkable (Table 1). While P4 showed some diastolic dysfunction, none of the patients had cardiomyopathy on echocardiography. There was no evidence of cardiac, neuropathic, ophthalmological, or hearing involvement.

### Muscle diagnostics: imaging findings and biopsy analyses

#### Imaging findings.

**Figure 2:**
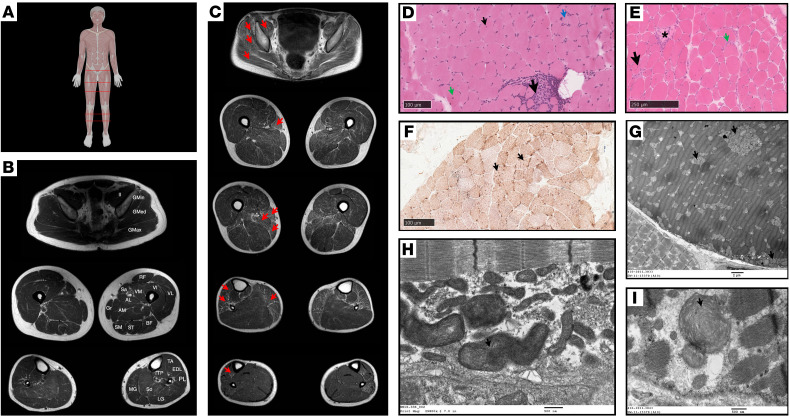
Muscle MRI, histopathology, and ultrastructural findings. (**A**) Anatomical reference. MRI scan positions are indicated with red lines on a human reference image. Figure 2A was created with BioRender.com. (**B**) Normal muscle MRI cross-sectional images showing anatomy of pelvic (top), thigh (middle), and lower leg (bottom) muscles. AL, adductor longus; AM, adductor magnus; BF, biceps femoris; EDL, extensor digitorum longus; Gmax, gluteus maximus; Gmed, gluteus medius; Gmin, gluteus minimus; Gr, gracilis; Il, iliacus; LG, lateral gastrocnemius; MG, medial gastrocnemius; PL, peroneus longus; RF, rectus femoris; Sa, sartorius; SM, semimembranosus; ST, semitendinosus; VI, vastus intermedius; TP, tibialis posterior; VL, vastus lateralis; VM, vastus medialis. (**C**) Axial muscle MRI images of P1 at age 17 years (proximal to distal) at pelvic, thigh, and calf levels showing diffuse fatty transformation (red arrows) greater in proximal muscles and more pronounced in the posterior thighs. (**D**) Quadriceps muscle biopsy from P2 at 6 years of age. H&E staining showed abnormal variation in fiber size, internal nuclei (small arrow), slight hypercontraction of fibers (green arrow), a focal area of cellularity possibly associated with necrosis (large black arrow), and slightly basophilic fibers (blue arrow). Scale bar: 100 μm. (**E**) Vastus lateralis muscle biopsy from P3 at 15 years of age. H&E staining shows abnormal variation in fiber size, necrotic fibers (*), increased number of internal nuclei (black arrow), increase in endomysial connective tissue, and endomysial cellularity (green arrow). Scale bar: 250 μm. (**F**) Combined cytochrome oxidase (COX) and succinic dehydrogenase (SDH) stains of quadriceps muscle from P2 at 6 years of age shows prominent mitochondria in several type 1 myofibers (arrows). Scale bar: 100 μm. (**G**) EM image of vastus medialis muscle biopsy from P1 at age 5 years and 10 months shows scattered clusters of morphologically abnormal mitochondria (arrows). Scale bar: 2 μm. (**H**) EM image of quadriceps muscle biopsy of P2 at 6 years of age shows morphologically abnormal, large mitochondria with whorled cristae (arrow). Scale bar: 500 nm. (**I**) Additional EM image from P1 shows large mitochondria with disoriented cristae with concentric arrangements (arrow). Scale bar: 500 nm.

A muscle ultrasound (P1) and muscle MRI (P1, P2, P3, P4) uncovered a distinctive pattern of muscle involvement correlating with the neuromuscular examination findings. The ultrasound revealed increased echogenicity of muscles in the upper and lower extremities (proximal > distal) and of the paraspinal muscles. The lateral gastrocnemius and soleus muscles were hypertrophied, while the medial gastrocnemius muscle was atrophied and markedly echogenic (Figure 2, A–C). Muscle MRI showed mild, diffuse fatty transformation that was greater in proximal muscles (all patients), slightly more pronounced in the posterior thigh compartment (P1) (Figure 2C), and affected more significantly the sartorius muscle (P1, P2, P3). In the lower extremities, there was hypertrophy of the soleus and gastrocnemius muscles, particularly the lateral gastrocnemius (P1, P2), and more selective fatty transformation of specific muscles including the medial gastrocnemius (all patients), tibialis anterior, and peroneus muscles (P1, P2) (Figure 2C).

#### Histopathological and ultrastructural muscle biopsy findings.

In all 4 patients, H&E-stained sections showed evidence of a myopathy with dystrophic features including variation in fiber size and an increase in internalized nuclei (Figure 2, D and E), as well as scattered degenerating/regenerating fibers and a mild-to-minimal increase in endomysial fibrosis (Figure 2, D and E). Foci of infiltrating immune cells, mainly macrophages (Figure 2D, large black arrow) were noted in all 4 cases. Upregulation of MHC-I was also noted, when assessed (P2, P3). Punctate material was present in a cytoplasmic distribution in mainly type I myofibers (Figure 2F). The material strongly stained for cytochrome c oxidase (COX), succinic dehydrogenase (SDH), or combined COX-SDH (Figure 2F) and thus corresponded to mitochondria. Electron microscopy (EM) was performed on P1 and P2 skeletal muscle and revealed morphologically abnormal mitochondria, often in clusters, enlarged and elongated with aberrant cristae ultrastructure (Figure 2, G–I).

#### Brain MRI findings.

**Figure 3:**
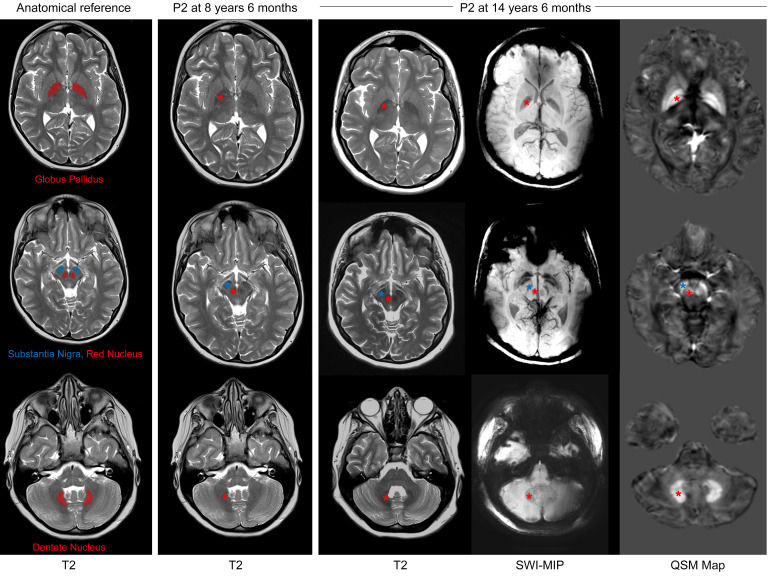
Brain MRI of P2 demonstrating evolving increased iron deposition in deep nuclei of the brain. Brain MRI of P2 performed at age 8 years 6 months shows normal anatomy and susceptibility signals. Brain MRI acquired at age 14 years 6 months shows increased, atypical-for-age susceptibility of bilateral globus pallidus (interna and externa with laminar sparing, upper row), substantia nigra (middle row), red nucleus (middle row), and dentate nucleus (lower row). The increased mineralization is evidenced as hypointense signal on T2 and SWI, and hyperintensity on QSM. The areas of interest are denoted by asterisks, with their color coding corresponding to the regions specified in the anatomical reference on the left. Of note, mild, diffuse cerebral and cerebellar volume reduction was also apparent when compared with the earlier scan. The left panel is displayed for anatomical reference.

Brain MRI scans (P1, P2, P3) did not reveal any significant structural or white matter abnormalities. Notably, 2 patients (P1, P2) presented with increased iron deposition, beyond what would typically be expected for their age, based on findings on T2, susceptibility-weighted imaging (SWI), and quantitative susceptibility mapping (QSM) sequences of the deep-brain nuclei (globus pallidus, substantia nigra, red nucleus, and dentate nucleus) (Figure 3). The increased iron deposition had likely developed gradually, as it was not evident on earlier brain MRI scans for P1 and P2 (Figure 3) (18, 19). Additional brain MRI findings included a single micro-hemorrhage in the left middle frontal gyrus (P1), right parietal white matter hyperintensities (P2), and mild, diffuse cerebral and cerebellar volume reduction in brain MRI compared with an earlier scan (P2).

### Biochemical and functional studies of CIAO1 variants identified in patients

#### The CIAO1 variants identified in patients cause protein instability and compromised biogenesis of multiple Fe-S proteins that acquire their clusters from the CIA complex.

**Figure 4:**
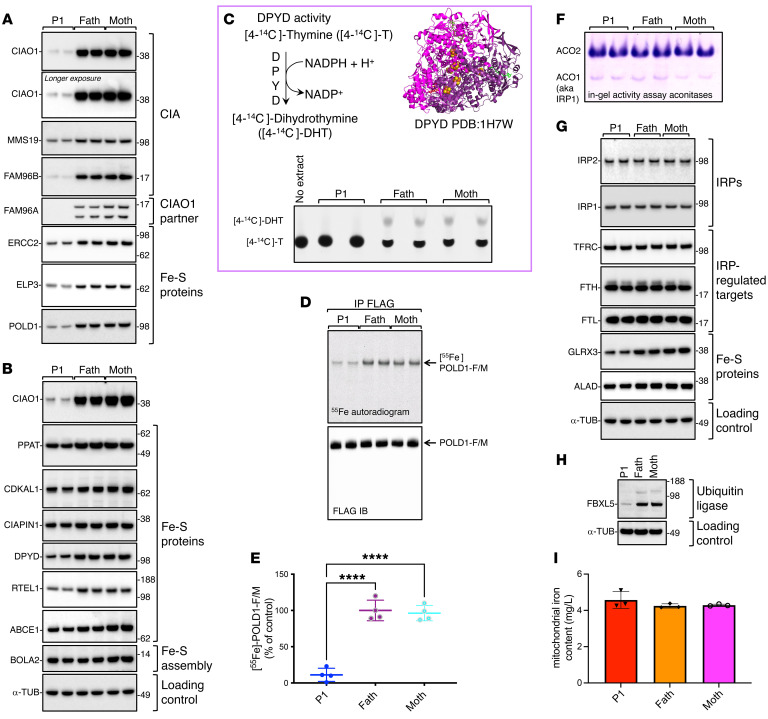
The *CIAO1* variants identified in P1 cause protein instability and compromised biogenesis of multiple Fe-S clients of the CIA complex. (**A** and **B**) Levels of the CIA components and Fe-S proteins in P1- and parent-derived fibroblasts (“Fath” and “Moth” correspond to father and mother of P1, respectively). Levels of FAM96A are also shown, along with the cytosolic iron and ISC chaperone BOLA2 (46). α-Tubulin (α-TUB) was included as a loading control and is presented again in panel **G**. To avoid reprobing of the same blotting membrane, the same lysates were run on adjacent wells on the gel shown in Supplemental Figure 4A, and α-tubulin was probed only once for the set of samples. (**C**) Top left corner shows the reaction catalyzed by DPYD. Top right corner is a ribbon representation of the crystal structure of DPYD (Protein Data Bank [PDB] ID: 1H7W), which assembles into a dimer containing a total of 8 [4Fe-4S] clusters. Bottom section shows DPYD-mediated conversion of [4-^14^C]-thymine to [4-^14^C]-dihydrothymine in lysates derived from P1 or control cells assayed by TLC and autoradiography. The reaction mix containing [4-^14^C]-T alone (no extract) was loaded to visualize the substrate (4-^14^C-thymine). (**D**) ^55^Fe incorporation into POLD1-FLAG/MYC expressed for 16 hours in P1 and parental fibroblasts. Anti-FLAG IB shows equal amounts of POLD1-F/M immunoprecipitated (**A–D**, *n* = 4 biological replicates). (**E**) Quantification by scintillation counter of ^55^Fe incorporated into POLD1-F/M. [^55^Fe]-POLD1-F/M levels in control cells (father of P1) were quantified and set to 100%. Values are expressed as a percentage of control and are given as the mean ± SEM. ****P < 0.0001, by 1-way ANOVA Šidák’s multiple-comparison test for P1 versus the father and P1 versus the mother. *n* = 4 biological replicates. (**F**) In-gel activity assays of cytosolic (ACO1) and mitochondrial (ACO2) aconitases in fibroblasts from P1 compared with control cells. (**G**) IBs to detect IRP1 and IRP2, TFRC, FTH, FTL, GLRX3, and ALAD on lysates from P1- and parent-derived fibroblasts. (**H**) Levels of FBXL5 in P1 and parental cells (**F** and **G**, *n* = 3 biological replicates). (**I**) Iron content in P1- and parent-derived mitochondria as assessed by ICP-MS (*n* = 3 biological replicates). No statistically significant difference was detected between experimental groups by 1-way ANOVA Šidák’s multiple-comparison test.

The amino acid residues substituted in the patients are completely conserved across different species (Supplemental Figure 2A), and structural analysis revealed that they are located on the short loops that interconnect the β-propeller domains (also known as blades) of CIAO1 (Supplemental Figure 2B), likely playing critical roles in proper folding and stability of the protein. In particular, the CIAO1 domain deleted in P1 (Phe250 through Leu339) is required to anchor CIAO1 to FAM96B in the overall architecture of the CIA complex (Supplemental Figure 2B). CIAO1 protein levels were profoundly diminished in cytosolic lysates from P1-derived fibroblasts compared with parental cells (Figure 4, A and B, and Supplemental Figure 3, A and B), with a concomitant loss of the known CIAO1-interacting partners MMS19 and FAM96B (Figure 4A and Supplemental Figure 3, A and B). Levels of the FAM96B paralogous protein FAM96A, which has been shown to interact with CIAO1 but not with MMS19 (20), were also decreased in P1-derived lysates compared with parental cells (Figure 4A). Following the loss of the 3 core components of the CIA complex, which specifically localizes to the cytosolic compartment of mammalian cells (Supplemental Figure 3, A and B), the levels of several Fe-S proteins that are known clients of the CIA machinery were decreased in lysates from P1 compared with parental cells (Figure 4, A and B). This observation is consistent with the reported instability of recipient Fe-S apoproteins when they fail to ligate their cofactors (21–24). Levels of the components of the de novo ISC assembly machinery, NFS1, HSC20, and HSPA9, which localize to both the cytosolic and mitochondrial compartments of mammalian cells (5, 7–12), were unchanged in lysates from P1 fibroblasts compared with parental cell lysates (Supplemental Figure 3, C and D). Functional assays revealed selective compromise of the biogenesis of Fe-S enzymes that are known clients of the CIA complex, namely dihydropyrimidine dehydrogenase (DPYD) (Figure 4C), which is involved in pyrimidine base degradation, and DNA polymerase delta catalytic subunit (POLD1), which is responsible for replication of the DNA lagging strand (Figure 4, D and E).

To assess the effect of the amino acid substitutions on protein stability and function, we used HeLa cells to generate and induce stable expression of the *CIAO1* variants identified in the patients. All variant CIAO1 proteins had diminished stability when compared with WT CIAO1 and reduced interactions with the CIA and the de novo ISC assembly components, as well as with recipient Fe-S proteins (Supplemental Figure 3E). Collectively, these findings strongly support that the identified variants are indeed pathogenic.

Consistent with the notion that the CIA machinery is a specialized complex responsible for the biogenesis of a large subset of cytoplasmic and nuclear Fe-S proteins but not all cytosolic enzymes, cytosolic aconitase 1 (ACO1, also known as IRP1) activity was unaltered in P1-derived lysates compared with parental cell lysates (Figure 4F), and there was no evidence of altered iron homeostasis (Figure 4, G and H, and Supplemental Figure 4, A–F) or mitochondrial iron overload (Figure 4I) in fibroblasts from P1 compared with parental cells.

**Figure 5:**
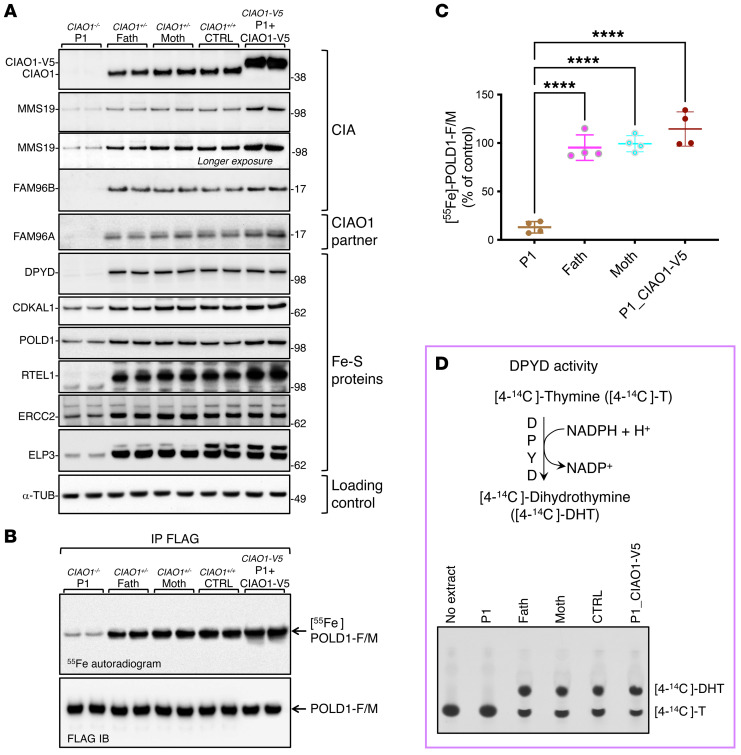
Lentivirus-mediated transduction of V5-tagged WT *CIAO1* in patient-derived cells reversed all the abnormalities caused by impaired function of the CIA machinery. (**A**) IBs to detect CIAO1, MMS19, FAM96B, FAM96A, and recipient Fe-S proteins (DPYD, CDKAL1, POLD1, RTEL1, ERCC2, and ELP3) on lysates from P1-derived (*CIAO1^–/–^*), parent-derived (*CIAO1^+/–^*), and control-derived (CTRL, *CIAO1^+/+^*) fibroblasts and from P1-derived fibroblasts after lentivirus-mediated restoration of WT *CIAO1* (*CIAO1-V5)* (*n* = 4 biological replicates). (**B**) Representative ^55^Fe incorporation into POLD1-FLAG/MYC transiently expressed in P1-derived, parent-derived, and control-derived (FC2-derived) fibroblasts and in P1-derived fibroblasts after lentivirus-mediated restoration of WT *CIAO1* (*CIAO1-V5)*. Anti-FLAG IB shows that equal amounts of POLD1-F/M were immunoprecipitated (*n* = 4 biological replicates). (**C**) Quantification of radioactive iron incorporated into POLD1-F/M as assessed by scintillation counter. [^55^Fe]-POLD1-F/M levels in control cells (father of P1) were quantified and set to 100%. Values are expressed as a percentage of control and are given as the mean ± SEM. ****P < 0.0001 by 1-way ANOVA Šidák’s multiple-comparison test for P1 versus the father, P1 versus the mother, and P1 versus P1_CIAO1-V5. *n* = 4 biological replicates. (**D**) Top section: schematic representation of the reaction catalyzed by the cytosolic Fe-S enzyme DPYD. Bottom section: DPYD-mediated conversion of [4-^14^C]-thymine to [4-^14^C]-dihydrothymine in lysates derived from P1, parental, or control fibroblasts (representing a fibroblast cell line harboring 2 WT copies of *CIAO1*, CTRL), and in P1- derived fibroblasts stably expressing *CIAO1-V5*, as indicated, assayed by TLC and autoradiography. The reaction mix containing the substrate of the reaction, [4-^14^C]-T without cell extract, was loaded as a negative control (no extract) to visualize the substrate (4-^14^C-thymine) by TLC (*n* = 3 biological replicates).

To unequivocally demonstrate that the phenotype reported in the patient cells was due to loss of function in *CIAO1*, we performed lentivirus-mediated transduction of C-terminally V5-tagged *CIAO1* in P1-derived fibroblasts to restore expression of the WT protein. We found that CIAO1-V5 fully reverted the abnormalities of the patient’s cells; specifically, it restored levels of the CIA components MMS19 and FAM96B and of FAM96A, and stabilized recipient Fe-S proteins that acquire their clusters from the CIA complex (Figure 5A). Moreover, functional assays demonstrated normalized radioactive iron incorporation into POLD1 in patient-derived cells upon reexpression of *CIAO1-V5* (Figure 5, B and C), along with full restoration of DPYD activity (Figure 5D).

**Figure 6:**
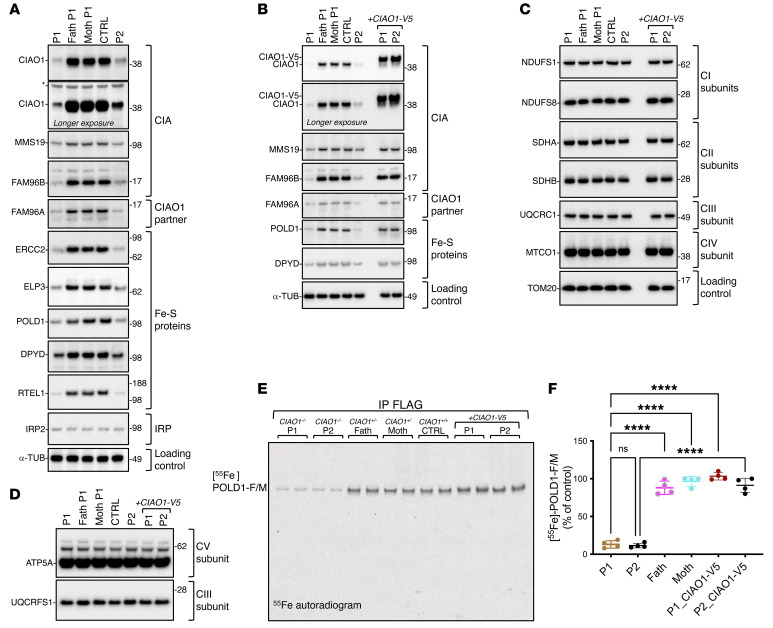
A second cell line derived from P2 demonstrates abnormal characteristics similar to those seen in P1 cells, and these defects in P2-derived cells are entirely reversed when the WT *CIAO1* gene is reintroduced. (**A**)SDS IBs were used to detect CIA components, FAM96A, and Fe-S recipient proteins (ERCC2, ELP3, POLD1, DPYD, and RTEL1) in fibroblasts from P1, his parents, a control cell line with 2 wild-type *CIAO1* copies (CTRL), and P2. α-Tubulin was used as a loading control (*n* = 3 biological replicates). (**B**) SDS IBs also detected CIA components, FAM96A, and Fe-S recipient proteins (POLD1, DPYD) in the same cell lines as in (**A**). Lysates from P1 and P2 cell lines transduced with V5-tagged wild-type *CIAO1* showed full restoration of CIA components and Fe-S recipient levels (*n* = 3 biological replicates). (**C**) SDS IBs to detect subunits of the mitochondrial respiratory chain complexes I (NDUFS1, NDUFS8), II (SDHA, SDHB), III (UQCRC1), and IV (MTCO1) in lysates from the cell lines presented in **B**. Levels of the mitochondrial marker TOM20 are shown as a reference for the loading control (*n* = 3 biological replicates). (**D**) SDS IBs to detect the mitochondrial respiratory chain subunits of complex V (CV) (ATP5A) and complex III (CIII) (UQCRFS1) in lysates from the cell lines presented in **B** and **C** (*n* = 3 biological replicates). (**E**) Representative ^55^Fe incorporation into POLD1-FLAG/MYC expressed in cell lines as presented the same cell lines presented in panels **B** and **C** (*n* = 4 biological replicates). (**F**) Quantification of radioactive iron incorporated into POLD1-F/M as assessed by scintillation counter. [^55^Fe]-POLD1-F/M levels in control cells (father of P1) were quantified and set to 100%. Values are expressed as a percentage of the control and shown as the mean ± SEM. ****P < 0.0001, by 1-way ANOVA Šidák’s multiple-comparison test for P1 versus the father, P1 versus the mother; P1 versus P1_CIAO1-V5 and P2 versus P2_CIAO1-V5. P1 versus P2 was not statistically significant (NS, *P* = 0.9991). *n* = 4 biological replicates.

A second primary fibroblast cell line derived from P2 was generated and biochemically characterized. Despite harboring only missense variants in CIAO1 (p.H302P/p.R65W), P2- derived fibroblasts exhibited a similar loss of CIAO1 protein as observed in P1-derived cells (Figure 6A), along with compromised stability of FAM96B, MMS19, FAM96A, and several cytoplasmic and nuclear Fe-S enzymes (Figure 6A). Lentivirus-mediated restoration of *CIAO1* expression in P2-derived fibroblasts restored the levels of MMS19 and FAM96B, as well as the stability of recipient Fe-S proteins (Figure 6B). Neither P1- nor P2-derived fibroblasts exhibited a significant mitochondrial defect (Figure 6, C and D, Supplemental Figure 5, A–I, and K, and Supplemental Figure 6, A–C), probably because of their dependency on glycolysis for energy production in the high-glucose media (4.5 g/L) of our experiments (25, 26), which may mask a secondary mitochondrial dysfunction in fibroblasts (vide infra for the muscle tissue results).

Functional assays in P2-derived fibroblasts revealed compromised biogenesis of Fe-S enzymes that depend on the CIA complex for cluster acquisition. Specifically, radioactive iron incorporation into POLD1 was severely impaired to an extent comparable to that observed in P1 (Figure 6, E and F). Lentivirus-mediated reexpression of WT *CIAO1* in P2 fibroblasts normalized ^55^Fe incorporation into POLD1 (Figure 6, E and F), confirming that loss of CIAO1 compromised ligation of the Fe-S cofactor in POLD1.

#### Patient’s skeletal muscle reveals compromise of CIA complex activity and abnormal mitochondrial morphology and function.

**Figure 7:**
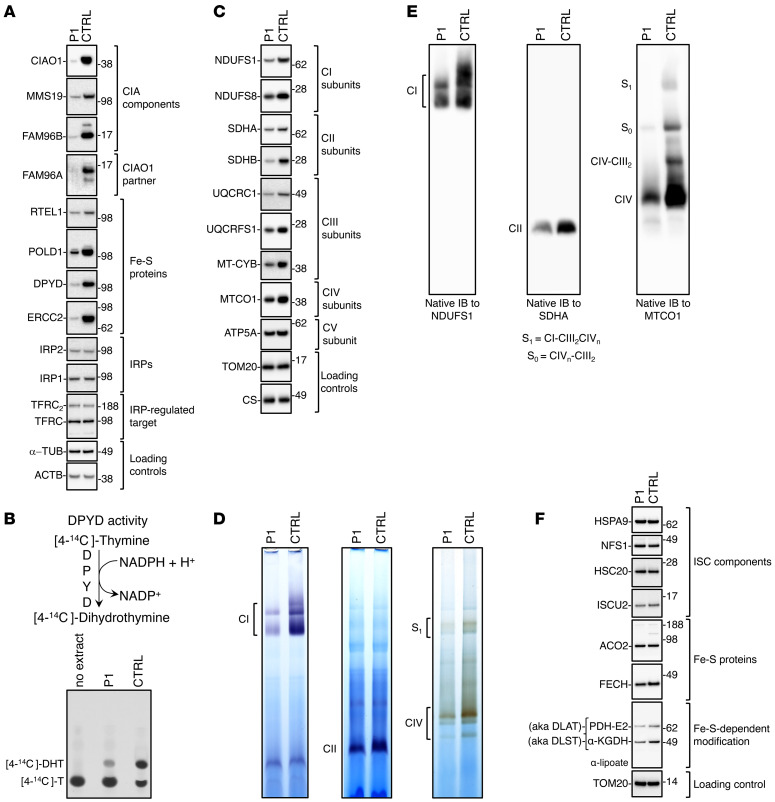
Mitochondrial dysfunction and compromised biogenesis of Fe-S recipients of the CIA complex in muscle from P1. (**A**) SDS IBs to detect the CIA components (CIAO1, MMS19, FAM96B) and FAM96A in P1 and control (*CIAO1^+/+^*) muscle tissue specimens. Levels of cytoplasmic and nuclear Fe-S proteins (RTEL1, POLD1, DPYD, ERCC2), of the IRPs IRP1 and IRP2, and of the IRP-regulated target TFRC were also assessed (TFRC_2_ designates dimeric TFRC). β-Actin (ACTB) and α-tubulin were included as references for even loading between samples. (**B**) Top panel illustrates the reaction catalyzed by the cytosolic Fe-S enzyme DPYD. Blot shows DPYD-mediated conversion of [4-^14^C]-thymine to [4-^14^C]-dihydrothymine in lysates derived from P1-derived and control-derived (*CIAO1^+/+^*; CTRL) muscle tissue specimens, assayed by TLC and autoradiography. The reaction mix containing the substrate of the reaction [4-^14^C]-T without cell extract was loaded as a negative control (no extract) to visualize the substrate (4-^14^C-thymine). (**C**) SDS IBs of lysates from isolated mitochondria to detect subunits of mitochondrial respiratory complex I (NDUFS1 and NDUFS8), complex II (SDHA, SDHB), complex III (UQCRC1, UQCRFS1, MT-CYB), complex IV (MTOC1), and complex V (ATP5A) in P1- and control-derived muscle tissue specimens. Levels of TOM20 and CS were included as a reference for the loading control. (**D**) In-gel activity assays of mitochondrial respiratory complexes I, -II, and -IV in P1- and control-derived muscle tissue specimens. (**E**) Native IBs of subunits of complex I (NDUFS1), complex II (SDHA), and complex IV (MTCO1) to assess the overall levels of fully assembled respiratory complexes. (**F**) SDS IBs of lysates from isolated mitochondria to detect components of the de novo ISC biogenesis pathway proteins HSPA9, NFS1, HSC20, and ISCU, the mitochondrial Fe-S enzymes ACO2 and FECH, and lipoylated PDH and α-KGDH complexes using an anti-lipoate antibody. Lipoylation is a posttranslational modification that depends on the Fe-S enzyme lipoic acid synthase LIAS (**A**–**F**, *n* = 2 biological replicates).

Given the consistent manifestation of skeletal muscle involvement in our patients with biallelic *CIAO1* variants, we decided to expand our biochemical analysis to include muscle biopsy material, which was available from P1. We found that the levels of CIAO1 and the other CIA components, MMS19 and FAM96B, were profoundly diminished in P1 skeletal muscle (Figure 7A), with concomitant loss of several cytoplasmic and nuclear Fe-S proteins (Figure 7A). Similar to observations in P1-derived fibroblasts, levels of iron-regulatory proteins (IRP1 and IRP2), and of the IRP-regulated membrane iron importer transferrin receptor (TFRC) were unaltered in P1-derived muscle tissue lysates compared with the control (Figure 7A), whereas DPYD activity was severely impaired (Figure 7B).

The EM data collected for P1 and P2 were indicative of mitochondrial ultrastructural abnormalities in the patients’ skeletal muscle (Figure 2, G–I); concomitantly, we observed compromised assembly and activities of the mitochondrial respiratory chain complexes. Specifically, the levels of subunits of complexes I, II, III and IV were all decreased in P1 muscle mitochondria compared with control lysates (Figure 7C), and the activities and levels of fully assembled complexes I, II, and IV were also reduced (Figure 7, D and E, respectively), pointing to functional and morphological mitochondrial dysfunction in the muscle secondary to *CIAO1* loss of function. To investigate whether a defect in mitochondrial respiration directly resulted from impairment of mitochondrial ISC biogenesis, we assessed levels of the mitochondrial Fe-S assembly machinery and found, notably, that the levels of most of the ISC biogenesis proteins were normal in P1 muscle samples compared with levels in controls, with only a minor decrease noted in ISCU (Figure 7F). Moreover, mitochondrial matrix Fe-S proteins, such as aconitase (ACO2), appeared unaffected. We noted a moderate reduction in the levels of ferrochelatase (FECH) (Figure 7F). The levels of lipoylated pyruvate dehydrogenase (PDH) and α-ketoglutarate dehydrogenase (α-KGDH) complexes, which rely on the mitochondrial Fe-S enzyme lipoic acid synthase (LIAS), were mildly diminished in P1 muscle samples compared with controls (Figure 7F).

## Discussion

The pathophysiological consequences of loss of function in *CIAO1*, the gene encoding a key component of the CIA complex, have thus far been unknown, as its role in the biogenesis of ISCs for nucleocytoplasmic Fe-S enzymes has been inferred solely on the basis of knockdown (KD) experiments in cultured cells (15, 20). We report here that biallelic pathogenic variants in *CIAO1* cause a disorder in humans with predominantly neuromuscular but also multisystemic manifestations. The amino acid sequence of CIAO1 is highly conserved across species from human to zebrafish, as are the residues mutated in the patients reported here (Supplemental Figure 2). The complementary studies in patient-derived cell lines and the biochemical characterization of the *CIAO1* variants confirmed the deleterious nature of the variants, which caused protein instability and compromised interaction with other CIA components and with Fe-S recipient proteins. Interestingly, in cell lines derived from patients, the chronic depletion of CIAO1 was associated with a concomitant decrease in the levels of its established interacting partners FAM96B, MMS19, and FAM96A. As previous reports on siRNA-mediated KD of *CIAO1* did not indicate a destabilization of FAM96B, MMS19, or FAM96A (20, 27), the loss of CIAO1-interacting partners was unexpected. Our findings highlight the distinction between the effect of an acute, temporary depletion of *CIAO1*, as achieved by siRNA-mediated KD, and a sustained loss, as observed in patient-derived cell lines, emphasizing the predominant involvement of the CIA components in a shared cellular pathway. Suboptimal levels of the CIA machinery led to compromised biogenesis of multiple Fe-S enzymes that play critical roles in genome maintenance (such as RTEL1), DNA replication (POLD1), tRNA modifications (ELP3 and CDKAL1), and purine and pyrimidine metabolism (PPAT and DPYD, respectively).

Patients presented with slowly progressing proximal and axial muscle weakness, respiratory insufficiency, elevated serum CK levels, and histologic muscle biopsy features consistent with a dystrophic myopathy characterized by abnormal mitochondrial morphology. Additional clinical manifestations of note included learning disabilities and neurobehavioral comorbidities, iron deposition in deep-brain nuclei, normocytic-to-macrocytic anemia in the context of normal levels of vitamins B12 and B9, and GI symptoms, indicating multisystemic consequences of diminished CIAO1 activity. The age of recognition of myopathic symptoms varied among our patients from early childhood to adolescence, even between the 2 patients who carried the same biallelic variants in *CIAO1* (P2 and P3). Three of 4 patients (P1, P2, and P4) remain ambulant, whereas P3 has progressed to being wheelchair bound at age 24.5 years, implying that the rate of progression of muscle weakness may vary as well. In this study, the carriers were not clinically affected, indicating that 1 functional copy of *CIAO1* is sufficient to sustain normal physiology, which is also consistent with the solely heterozygous variants found in the GnomAD database. The *CIAO1* variants identified in our patients resulted in only a partial loss of function, as global KO is likely to be incompatible with life. Thus far, only genetic ablation of MMS19 has been attempted in mice, leading to preimplantation death (13).

Interestingly, the pattern of brain iron deposition observed in the CIAO1-deficient patients is reminiscent of that observed in patients with neurodegeneration with brain iron accumulation (NBIA), a clinically and genetically heterogenous group of disorders affecting children and adults (28). NBIA is often first suspected when increased basal ganglia iron is observed on brain MRI (28). Abnormal accumulation of iron in the brain has been identified in several neurodegenerative conditions, yet the precise role of iron overload in disease pathology remains uncertain and the underlying mechanism largely uncharacterized (28, 29). Our patients do not present with profound neurological deficits, and they therefore differ significantly in clinical course from patients with classical NBIA.

Our studies of patient-derived fibroblasts and muscle samples revealed that cellular iron homeostasis was maintained upon loss of CIAO1 due to the counterbalancing of 2 opposing regulatory axes that control IRP2 protein levels (see Supplemental Figure 4F). Specifically, we propose that the absence of CIAO1 disrupts the normal turnover of IRP2 by reducing the levels of its ubiquitin ligase, FBXL5. Although the loss of FBXL5 would typically result in elevated IRP2 levels, the concomitant absence of both CIAO1 and FAM96A compromises the stabilization of IRP2 in patient-derived cells. This unexpected equilibrium of opposing regulatory mechanisms that control IRP2 degradation and stabilization ensures the preservation of iron homeostasis and the maintenance of normal levels of mitochondrial iron content. As a result, the CIAO1 disorder appears to be distinct from conditions arising from the loss of function in early-acting ISC biogenesis factors, which are typically characterized by mitochondrial iron overload (16, 30). Although the MRI-detected brain iron deposition in our cohort of patients and our findings of rebalanced iron-regulatory effects may seem contradictory, we believe they can be reconciled by our interpretation that brain iron overload in our patients is probably secondary to neurodegeneration induced by impaired activity of the CIA machinery and not directly linked to altered cellular iron homeostasis.

The broad phenotypic manifestation of loss of function in *CIAO1* underscores the critical role of the CIA machinery in delivering Fe-S cofactors to numerous essential nuclear and cytoplasmic Fe-S enzymes involved in all aspects of DNA metabolism, tRNA modification, and protein translation. Given the ubiquitous nature of these processes, it is likely that the spectrum of *CIAO1*-related disorders may vary and expand as new pathogenic variants are ascertained, depending on the specific effect that the amino acid substitutions have on protein stability and function.

Loss of function in several components of the Fe-S biogenesis pathway has been linked to multiple rare human conditions that manifest with different patterns of systemic or tissue-specific involvement (16). Relevant examples are the multiple mitochondrial dysfunctions syndromes (OMIM #605711, #614299, #615330, #616370, #617613, #617954, #620423), which manifest as severe autosomal recessive disorders of systemic energy metabolism, resulting in muscle weakness, respiratory failure, severely impaired neurologic development, lactic acidosis, and early death (16). However, several studies have also identified distinctive tissue-specific manifestations as the main characteristic in a number of disorders caused by variants in the Fe-S biogenesis components, including the sideroblastic anemia caused by loss of function in glutaredoxin 5 (*GLRX5*) (OMIM #616860) (31, 32). Additionally, a subset of muscle-specific disorders has also been documented, including the myopathy with lactic acidosis due to aberrant splicing of the *ISCU* transcript (OMIM #255125) (33). This condition leads to a muscle-specific loss of Fe-S proteins, along with mitochondrial iron accumulation, causing symptoms such as poor endurance, muscle cramps, lactic acidosis and severe episodes of myoglobinuria (33, 34). While global KO of *ISCU* in mice results in early embryonic lethality (35), the intronic variant identified in patients allows an aberrant splicing pattern of *ISCU* that leaves some residual function, potentially providing insights into the muscle-specific phenotype of the disease (33, 36).

*FDX2*, encoding a small protein involved along with FDXR in donating electrons to nascent ISCs, was the second gene in the Fe-S biogenesis pathway to be linked to a mitochondrial myopathy (OMIM #251900) (37). Following the first report in 2014 of a single patient who presented with adolescent onset of autosomal recessive mitochondrial myopathy (37), a second report of a different variant 5 years later broadened the spectrum of the *FDX2*-related disorder in 6 patients from 2 Brazilian families (38). This new variant was linked to early onset of neurological symptoms, optic atrophy, and myopathy characterized by recurrent episodes of cramps, myalgia, and muscle weakness (38). Sensory-motor axonal neuropathy and leukoencephalopathy with reversible white matter changes were also shown to be part of the extended phenotype (38).

The muscle histopathological and ultrastructural features of the CIAO1-deficient patients demonstrate a combination of unique characteristics, including mixed moderate myopathic and dystrophic changes. Additionally, strikingly large and morphologically abnormal mitochondria were observed, whereas histopathology lacked classic findings commonly seen in mitochondrial myopathies such as COX-negative fibers and SDH deficiency. This distinction sets the CIAO1 myopathy apart from the classical mitochondrial Fe-S–associated myopathies like the ISCU and FDX2 myopathies. While the CIAO1-related muscle pathology does not fit the conventional criteria of a mitochondrial myopathy, it exhibits a discernible mitochondrial dysfunction, as demonstrated by histological, ultrastructural, and functional assessments. We speculate that the mitochondrial dysfunction in CIAO1-deficient muscle might be secondary to the loss of several nucleocytoplasmic Fe-S enzymes that depend on CIA for function. Sufficient levels of those enzymes are critical to meet the cellular needs for transcriptional and translational activities. Therefore, this impairment becomes particularly notable in muscle tissue, known for its high protein turnover rates (39). Interestingly, we observed the enlarged mitochondria mainly in type 1 myofibers (Figure 2F, type 1 = darker myofibers), which are known to be rich in mitochondria and to rely on aerobic metabolism, further supporting the idea of a cell-specific threshold requirement of the CIA machinery for proper function.

We observed a more pronounced decrease in the levels of respiratory chain complexes compared with mitochondrial matrix proteins (e.g., ferrochelatase, aconitase, or ISC biogenesis components), despite no significant change in mitochondrial mass. Several factors may contribute to this disparity, including alterations in mitochondrial morphology observed in P1 and P2, which could potentially affect the assembly and architecture of the membrane-embedded respiratory complexes. Additionally, defective assembly of the multisubunit respiratory complexes due to inadequate energy production, potential differences in turnover rates of mitochondrial proteins, or activation of compensatory mechanisms to maintain protein levels could also play a role. Although we cannot definitively pinpoint the primary cause, we have ruled out pathogenic variants or deletions in mitochondrial DNA (mtDNA). It is plausible that secondary mitochondrial dysfunction may be present in other organs of the CIAO1-deficient patients, as our analysis was limited to muscle tissue due to its accessibility for sampling. Overall, these results uncover how a disruption in the cytoplasmic Fe-S assembly machinery precipitates a secondary mitochondrial defect, within the framework of an otherwise intact Fe-S biogenesis pathway within mitochondria.

Although the exact phenotypic spectrum of the *CIAO1*-related disorder remains to be fully defined and will likely become clearer as more patients are identified, our findings contribute to a better understanding of the role of CIAO1 in the biogenesis of ISCs for nucleocytoplasmic Fe-S enzymes. Additionally, our study defines the essential role of CIAO1 for human health and offers insights into a previously uncharacterized multisystem disorder.

## Methods

### Sex as a biological variable.

Sex was not considered as a biological variable in this study. The patient cohort in this study primarily consisted of females and only 1 male.

### Recruitment and sample collection.

We studied 4 unrelated patients with biallelic *CIAO1* variants — P1, P2, P3, and P4 — aged 17, 14, 25, and 59 years, respectively. All patients were followed in specialized neuromuscular clinics because they were experiencing muscle weakness of unknown etiology. P1 originated from the United States and was referred to the NIH by his neurologist. P2 originated from the United Kingdom and was identified through the Matchmaker Exchange platform (40), and P3 and P4 , from the United Kingdom and Serbia, respectively, were identified through the MYO-SEQ program (41). Medical history and clinical evaluations, including muscle and brain MRI and muscle biopsies, were performed as part of the diagnostic efforts as standard diagnostic procedures. Laboratory tests, muscle biopsy histology slides, and electron microscopy (EM) images/reports were independently reviewed. The patients’ *CIAO1* variants were identified by whole-exome sequencing performed on whole-blood DNA obtained using standard procedures. Samples for research-based testing, including blood (all patients), skin fibroblasts (P1, parents of P1, and P2), and muscle tissue (P1) were obtained via standard procedures. The muscle biopsy tissue was mounted in the gum guar oriented vertically, frozen in precooled isopentane (2-methyl butane), and stored at –80°C before testing.

### Exome-, genome-, and RNA-Seq.

P1 whole-exome sequencing and analysis were performed using the Agilent Clinical Research Exome kit and the Illumina HiSeq 2000 sequencing system with 100 bp paired-end reads and analyzed for sequence variants using a custom-developed analysis tool (Xome Analyzer, GeneDx). For P2, P3, and P4, whole-exome sequencing and data processing were performed by the Genomics Platform at the Broad Institute of MIT and Harvard with a TWIST exome kit (P2) or with an Illumina Nextera (P3 and P4) then and sequenced (150 bp paired reads) to cover greater than 80% of targets at 20× and a mean target coverage of greater than 100×. Exome-sequencing data were processed through a pipeline based on Picard, and mapping was done using the BWA aligner to the human genome build 38. Variants were called using the Genome Analysis Toolkit (GATK) HaplotypeCaller package, version 3.5.

P1 human whole-transcriptome sequencing of fibroblasts was performed by the Genomics Platform at the Broad Institute of MIT and Harvard. The transcriptome product combines poly(A) selection of mRNA transcripts with a strand-specific cDNA library preparation, with a mean insert size of 550 bp. Libraries were sequenced on the HiSeq 2500 platform to a minimum depth of 50–75 million STAR-aligned reads. ERCC RNA controls are included for all samples, allowing additional control of variability between samples.

mtDNA was analyzed and was negative; mtDNA single nucleotide and small indel variants were called from exome-sequencing data using the MToolBox pipeline (42) and large mtDNA deletions were called by MitoSAlt (43).

### Sashimi plots.

Bam files were generated using the GTEXv10 pipeline (https://github.com/broadinstitute/gtex-pipeline) and aligned using the reference genome GRCh38 (Gencode, version 39). Sashimi plots were generated using ggSashimi and a minimum splice junction threshold of 10 reads set (44). Control sample plots, when not separated, represent the mean junction reads of 3 aggregate samples.

### Cell culture methods.

Dermal fibroblasts isolated from skin biopsies were grown in DMEM (4.5 g/L glucose), 2 mM glutamine, 10% FBS (Life Technologies, Thermo Fisher Scientific), and 1% penicillin/streptomycin (Life Technologies, Thermo Fisher Scientific) in 5% CO_2_ at 37°C.

### Lentivirus-mediated transduction of CIAO1-V5 in patient-derived fibroblasts.

Patient-derived fibroblasts were engineered to stably express C-terminally V5-tagged *CIAO1* by lentivirus-mediated transduction with pLENTI6.2/V5-DEST (Invitrogen, Thermo Fisher Scientific). The ViraPower Lentiviral Expression System (Invitrogen, Thermo Fisher Scientific) was used to produce viral particles harboring *CIAO1-V5* under the control of a CMV promoter, according to the manufacturer’s instructions. Briefly, pLENTI6.2/*CIAO1-V5* was cotransfected with the ViraPower Packaging Mix into HEK293T cells. The lentiviral stock collected 36 hours after transfection was used to transduce P1- and P2-derived fibroblasts. Stable clones were established after 6 days of selection with blasticidin. Expression levels of *CIAO1*-V5 were assessed by Western blotting.

### Site-directed mutagenesis and expression of CIAO1 variants in HeLa cells.

Point mutations and deletion into *CIAO1* were introduced using the QuikChange II site-directed mutagenesis kit (Agilent Technologies) following the manufacturer’s instructions. All clones were verified for insertion of the desired mutation by Sanger sequencing at Eurofins USA. Stable cell lines expressing WT CIAO1-V5 or the variants identified in the patients were generated by subcloning the *CIAO1* ORF into pLENTI6.2/V5-DEST (Invitrogen, Thermo Fisher Scientific). The ViraPower Lentiviral Expression System (Invitrogen, Thermo Fisher Scientific) was used to produce viral particles according to the manufacturer’s instructions. Briefly, pLENTI6.2/CIAO1-V5 was cotransfected with the ViraPower Packaging Mix into HEK293T cells. The lentiviral stock collected 36 hours after cotransfection was used to transduce HeLa cells. Stable clones were established after 6 days of selection with blasticidin. Expression levels of CIAO1-V5 WT and variant proteins were assessed by Western blotting.

### Subcellular fractionation into cytosol and mitochondria and immunoprecipitation experiments.

Subcellular fractionation into cytosol and intact mitochondria was done as previously described (5, 22, 23). Briefly, mitochondria from patient-derived fibroblasts or HeLa cell pellets (~10^9^ cells) were isolated from the cytosolic fractions after cell permeabilization with a buffer containing 0.1% digitonin in 210 mM mannitol, 20 mM sucrose, and 4 mM HEPES. The pellets after centrifugation at 700*g* for 5 minutes contained mitochondria, which were isolated by differential centrifugation and solubilized in lysis buffer I containing 50 mM Bis-Tris, 50 mM NaCl, 10% w/v glycerol, 0.001% Ponceau S, 1% lauryl maltoside, pH 7.2, and protease inhibitors.

The supernatants after the centrifugation at 700*g* containing soluble proteins were spun down at 21,000*g* for 20 minutes. The supernatant after the centrifugation was supplemented with a 1:1 volume (v/v) of a buffer containing 25 mM Tris, 200 mM NaCl, 1 mM EDTA, and 1% NP-40 (pH 7.4) to obtain a final protein concentration of approximately 1 μg/μL. Total cytosolic proteins (500 mg) were used for the immunoprecipitations (IPs) of WT CIAO1-V5 and variants using agarose anti-V5 beads (Abcam, ab1229). Equilibrated anti-V5 agarose beads were added to the lysates and incubated for 2 hours at room temperature (RT). Beads were extensively washed with lysis buffer, and bound proteins were eluted with Tris-glycine, pH 2.8, for 10 minutes at RT. The eluates were analyzed by SDS-PAGE and immunoblotting.

### Subcellular fractionation from muscle tissue sample.

Snap-frozen muscle tissue specimens were ground to powder with mortar and pestle. Four volumes of extraction buffer (5 mM MOPS, pH 7.5, supplemented with 250 mM sucrose, 1 mM EDTA, and 0.1% ethanol) were added per gram of tissue, and samples were extracted by 2 sets of dounce strokes (20 strokes) in a prechilled dounce homogenizer, followed by incubation on ice for 10 minutes. The homogenate was centrifuged at 1,000*g* for 10 minutes. Next, the supernatant was transferred into a new tube, and the pellet containing nuclei was washed with dilution buffer (5 mM MOPS, pH 8.0, supplemented with 1 mM EDTA and 0.1% ethanol), subsequently lysed in RIPA buffer, and sonicated for the extraction of nuclear proteins. The supernatant after the first centrifugation step at 1,000*g* was spun at 2,000*g* for 10 minutes. The supernatant after the centrifugation at 1,000*g* was spun at 25,000*g* for 20 minutes, and the pellet, containing a mixture of mitochondria, lysosomes, peroxisomes and ER membranes, was subjected to further purification by density gradient in OptiPrep Density Gradient Medium (MilliporeSigma, D1556) diluted to the final required concentration in 5 mM MOPS (pH 8.0) containing 1 mM EDTA and 0.1% ethanol. The pellets containing light mitochondria were resuspended in 450 mL of 22.5% OptiPrep Density Gradient Medium and layered between 200 mL of the 27.5% OptiPrep solution (at the bottom) and 200 mL of the 20% OptiPrep solution (on top). The gradient was centrifuged at 100,000*g* for 1.5 hours. Mitochondria sedimented at the 22.5%–27.5% interface and were lysed in 1.25× buffer I (50 mM BisTris, 50 mM NaCl, 10% w/v glycerol, 0.001% Ponceau S, 1.2% Lauryl maltoside, pH 7.2, protease and phosphatase inhibitors). The supernatant after centrifugation at 21,000*g* for 15 minutes was saved as mitochondrial lysates.

### DPYD activity assay.

DPYD activity was determined by thin-layer chromatography (TLC) according to a previously described protocol (14, 15), with the following modifications: cytosolic cell lysates containing 150 μg proteins isolated from patient-, parent-, and control-derived fibroblasts or from muscle tissue lysates, as specified in the main text and figure legends, were applied to 50 μL of a reaction mix containing 25 mM Tris-HCl (pH 7.5), 0.1% digitonin, 2.5 mM MgCl_2_, 2 mM DTT, 10 μM [4-^14^C]-thymine (0.1 mCi/mL, Moravek), and 10 mM NADPH. After 4 hours of incubation at 32°C, the reaction was stopped by addition of 10 μL perchloric acid (10% v/v). Reaction mixtures were centrifuged at 20,000*g* for 5 minutes, and the supernatants were analyzed by TLC.

### Blue native PAGE analyses of mitochondrial respiratory complexes.

The Native PAGE Novex Bis-Tris gel system (Thermo Fisher Scientific) was used to assess activities and levels of mitochondrial respiratory chain complexes, with the following modifications: only the Light Blue Cathode Buffer was used; 20 mg membrane protein extracts were loaded/well; and electrophoresis was performed at 150 V for 1 hour and 250 V for 2 hours.

### Complex I, complex II, and complex IV in-gel activity assays and native immunoblots.

In-gel complex I, complex II, and complex IV activity assays were performed as previously described (5, 22, 23). For complex I activity, after resolution of the respiratory complexes by blue native PAGE (BN-PAGE), the gel was incubated with 0.1 M TrisCl, pH 7.4, containing 1 mg/mL nitrobluetetrazolium chloride (NBT) and 0.14 mM NADH at RT for 30–60 minutes. For complex II, detection of succinate CoQ-reductase activity (SQR) (CoQ-mediated NBT reduction) was performed by incubating the gel for 30 minutes with 84 mM succinate, 2 mg/mL NBT, 4.5 mM EDTA, 10 mM KCN, 1 mM sodium azide, and 10 mM ubiquinone in 50 mM PBS, pH 7.4. For complex IV, the gel was incubated in 50 mM phosphate buffer, pH 7.4, containing 1 mg/mL DAB and 1 mg/mL cytochrome c at RT for 30–45 minutes.

For the native immunoblots (IBs), PVDF was used as the blotting membrane. The transfer was performed at 25 V for 4 hours at 4°C. After transfer, the membrane was washed with 8% acetic acid for 20 minutes to fix the proteins and then rinsed with water before air drying. The dried membrane was washed 5–6 times with methanol (to remove residual Coomassie Blue G-250), rinsed with water, and then blocked for 2 hours at RT in 5% milk before incubation with the desired antibodies diluted in 2.5% milk overnight at 4°C. In order to avoid stripping and reprobing of the same membrane, which might allow detection of signals from the previous IBs, samples were loaded and run in replicates on adjacent wells of the same gel and probed independently with different antibodies.

### Aconitase in-gel activity assay.

The aconitase activity assay was performed as previously described (45).

### Inductively coupled plasma mass spectrometry.

Iron content in the patient- and parent-derived fibroblasts was determined by ICP-MS (Agilent Technologies, model 7900). Concentrated trace metal–grade nitric acid (200 μL, Thermo Fisher Scientific) was added to isolated mitochondria, and the organelles were digested overnight at 85°C. Each sample was analyzed by ICP-MS after dilution with 3.8 mL deionized water.

### Immunoblotting.

Antibodies in this study were used at 1:1,000 dilution unless otherwise specified and were as follows: anti-IRP1 antibody was prepared against purified human IRP1 and used at 1:5,000 dilution. Anti-IRP2 antibody was prepared against a peptide covering the amino acid residues 137–209 of human IRP2 and used at 1:2,000 dilution. Anti-ACO2 rabbit polyclonal antibody was raised against the synthetic peptide YDLLEKNINIVRKRLNR. Anti-TFRC antibody was from Thermo Fisher Scientific. Anti–ferritin H (anti-FTH), -FTL, -NDUFS1, -NDUFS8, -NDUFV1, -SDHA, -SDHB, -MTCO1, -MTCO2, -UQCRC1, UQCRC2, -UQCRFS1, -ATP5A, –MT-CYB, –MFN1/-2, and total OXPHOS (complex V, ATP5A subunit; complex IV, COXII subunit; complex III, UQCRC2 subunit; complex II, SDHB; complex I, NDUFB9 subunit) antibodies were from Abcam. Anti-CIAO1, -NFS1, and-FBXL5 (catalog sc-54364, lot A1408) were from Santa Cruz Biotechnology. Anti-tubulin, –β-actin, -HSC20, -HSPA9, -FAM96A, and -ALAD were from MilliporeSigma. Anti-MMS19, -FAM96B, -ERCC2, -ELP3, -POLD1, -PPAT, -CDKAL1, -CIAPIN1, -GLRX3, -DPYD, -RTEL1, -ABCE1, -TOM20, -FECH, -MFN1, and –citrate synthase (CS) were from Proteintech. Anti-BOLA2 was from Bethyl Laboratories. Anti-lipoate antibody was from EMD Millipore. Anti-OPA1 was from BD Biosciences.

### Radiolabeling experiments.

The ^55^Fe incorporation assays were performed essentially as previously described (5, 22), with minor modifications. Patient- and parent-derived cell lines, as indicated, were grown in the presence of 1 μM ^55^Fe-transferrin (TF) for 5–7 days. Transient transfection of C-terminally FLAG/MYC-tagged POLD1 for 16 hours was followed by subcellular fractionation. Cytosolic extracts were subjected to IP with anti-FLAG agarose beads to immunocapture POLD1-FLAG. Samples collected after competitive elution (with 3× FLAG peptide at 100 mg/mL) were run on a native gel, followed by autoradiography.

Alternatively, ^55^Fe incorporation into POLD1-FLAG/MYC was measured by scintillation counting of M2 FLAG beads (MilliporeSigma, A2220) after immunoabsorption of POLD1-FLAG/MYC, followed by extensive washes with buffer I. The background, corresponding to ^55^Fe measurements of eluates after anti-FLAG IPs on cytosolic extracts from cells transfected with the empty vector, was subtracted from each reading.

### Statistics.

Where applicable, pairwise comparisons between 2 groups were analyzed using the 2-tailed, unpaired Student’s *t* test. Significance for multigroup comparisons was analyzed with 2-way ANOVA followed by Šidák’s multiple-comparison test. All tests were performed with GraphPad Prism 9 (GraphPad Software), and data are expressed as the mean ± SD or SEM, as specified in the figure legends. A *P* value of less than 0.05 was considered significant.

### Study approval.

Written informed consent and age-appropriate assent for research studies and procedures were obtained from the patients. Ethics approval was obtained for P1 via protocol 12-N-0095, approved by the NIH IRB; for P2 via the Great Ormond Street Hospital Research Ethics Committees GOSH 00/5802; and for both P3 and P4 via the National Research Ethics Service (NRES) Committee North East–Newcastle and North Tyneside 1 (reference 19/NE/0028).

### Data availability.

All data needed to evaluate the conclusions of this study are present in the main text and supplemental materials. Therefore, all data are readily available to be shared with the appropriate data-sharing agreements. There are no exceptions to the sharing of data, materials, or software programs. The next-generation sequencing data have been submitted to the following public databases: RNA-Seq data are available in the GREGoR database (accession ID: phs003047). DNA-Seq data are accessible from the Broad Institute Center for Mendelian Genomics (Broad CMG) dbGaP database (accession ID: phs001272). Values for all data points in graphs presented in Figure 4, E and I, Figure 5C, and Figure 6F are provided in the Supporting Data Values file.

## Author contributions

NM, RO, ITZ, AT, S Donkervoort, VS, TAR, CGB, and FM conceived of and designed the study. NM conducted experiments. NM, RO, ITZ, AT, S Donkervoort, PM, JM, TW, SV, SP, DK, S Sudhakar, ARF, S Silverstein, GD, LP, S DiTroia, CG, YH, CS, AS, VS, FM, TAR, and CGB acquired data. NM, RO, ITZ, AT, S Donkervoort, PM, JM, TW, SV, SP, DK, S Sudhakar, ARF, S Silverstein, GD, LP, S DiTroia, CG, YH, CS, AS, VS, FM, TAR, and CGB analyzed data. NM and TAR provided reagents. NM and RO wrote the original draft of the manuscript. NM, RO, VS, FM, TAR, and CGB provided critical revisions to the manuscript. All authors reviewed and approved the final version of the manuscript. The authorship order among the co–first authors was determined to reflect their substantial contribution to the study, while acknowledging varying degrees of involvement in the collaborative work.

## Supplementary Material

Supplemental data

Supporting data values

## Figures and Tables

**Table 1:**
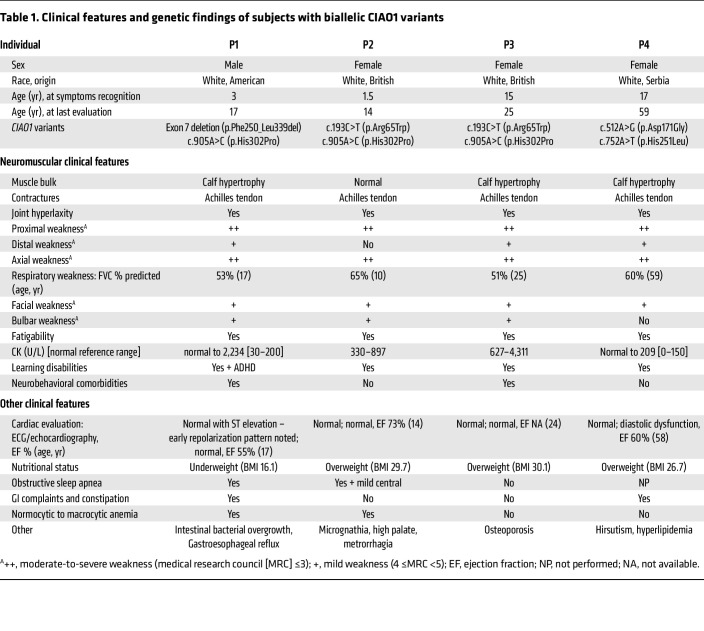
Clinical features and genetic findings of subjects with biallelic CIAO1 variants

## Version 1 06/17/2024

Electronic publication
